# Plasma and tissue concentrations of 2 g prophylactic cefazolin prior to lower extremity surgery

**DOI:** 10.1128/aac.00494-24

**Published:** 2024-05-21

**Authors:** Robin Eelsing, Diederick Penning, Marloes Vos-van der Meer, Caspar J. Hodiamont, Ron A. A. Mathôt, Tim Schepers

**Affiliations:** 1 Department of Surgery, Amsterdam UMC location University of Amsterdam, Amsterdam, the Netherlands; 2 Amsterdam Movement Sciences, AMS - Musculoskeletal Health, Amsterdam, the Netherlands; 3 Pharmacy laboratory, Amsterdam UMC location University of Amsterdam, Amsterdam, the Netherlands; 4 Department of Medical Microbiology, Amsterdam UMC location University of Amsterdam, Amsterdam, the Netherlands; 5 Hospital Pharmacy and Clinical Pharmacology, Amsterdam UMC location University of Amsterdam, Amsterdam, the Netherlands; Providence Portland Medical Center, Portland, Oregon, USA

**Keywords:** cefazolin, antibiotic prophylaxis, lower extremities, surgical site infection

## Abstract

Surgical site infections (SSIs) are among the most clinically relevant complications and the use of prophylactic cefazolin is common practice. However, the knowledge about the pharmacological aspects of prophylactic cefazolin in the lower extremities remains limited. In this prospective cohort, a sub-study of the WIFI-2 randomized controlled trial, adults between 18 and 75 years of age who were scheduled for implant removal below the level of the knee and randomized for cefazolin, was included. A maximum of two venous plasma, target-site plasma, and target-site tissue samples were taken during surgery. The primary outcomes were the cefazolin concentrations in venous plasma, target-site plasma, and target-site tissue. A total of 27 patients [median (interquartile range) age, 42 (29–59) years; 17 (63%) male] with 138 samples were included in the study. A minimum of 6 weeks follow-up was available for all patients. The mean (SD) venous plasma, target-site plasma, and target-site tissue concentrations were 36 (13) µg/mL, 29 (13) µg/mL, and 28 (13) µg/g, respectively, and the cefazolin concentrations between the different locations of surgery did not differ significantly in both target-site plasma and target-site tissue (*P* = 0.822 and *P* = 0.840). In conclusion, 2 g of prophylactic cefazolin demonstrates adequacy in maintaining coverage for a duration of at least 80 minutes of surgery below the level of the knee, significantly surpassing the MIC_90_ required to combat the most prevalent microorganisms. This study represents the first of its kind to assess cefazolin concentrations in the lower extremities by examining both plasma and tissue samples in this magnitude.

## INTRODUCTION

In surgery, surgical site infections (SSIs) are among the most clinically relevant complications. Both superficial and deep infections negatively affect the functional outcome following surgery and increase morbidity (1, 2).

Within (orthopedic) trauma surgery, implants are frequently involved, which makes treatment of infections more complex, and therefore, the clinical relevance of an SSI increases (3, 4). The frequency of SSI increases when surgery is performed at a more distal location in the body. In lower extremities, the rate of infection ranges from 1.3% to 10% above the level of the knee and from 12% to 25% below the level of the knee (5–10).

To reduce the risk of infection, antibiotic prophylaxis is widely used and is therefore part of the protocolled care for surgery using implants (11–14). First-generation cephalosporins are recommended and frequently used because of their broad-spectrum effect and low costs (11, 15). In the Netherlands, the recommended dose of cefazolin is 2 g for prophylaxis (11). However, the literature regarding recommendation of dosage is limited.

The dose of prophylactic antibiotics needs to result in concentrations exceeding the minimum inhibitory concentration (MIC) of the microorganism (16). The antibiotic concentration is usually measured in plasma, although SSIs occur in the subcutaneous tissue (17). Therefore, assessment of antibiotic concentrations at this target site could be of interest, because this may provide more insight into the clinical effectivity of the antibiotic. It is important to realize the concentrations in plasma and subcutaneous tissue will differ. Distribution of drugs is not homogenous in the body and antibiotic concentrations are lower in more distal parts of the limbs compared to proximal parts (18). Literature about antibiotic concentrations in foot and ankle is limited, especially for subcutaneous tissue concentrations. The primary aim of this study is to measure cefazolin concentrations at different locations below the level of the knee and to compare the measured target-site tissue/plasma as well as the venous plasma concentrations. Additionally, these concentrations will be compared with the MIC of the most common microorganisms causing an SSI, and an analysis to find possible predictors of the cefazolin concentration will be performed.

## MATERIALS AND METHODS

### Study design and setting

This was a single-center, prospective, observational pharmacokinetic study and a sub-study of the randomized controlled trial called the WIFI-2 study (19). All patients were admitted for implant removal below the level of the knee.

### Participants/study subjects

The aim was to include 24 consecutive patients receiving 2 g of prophylactic cefazolin. Inclusion criteria were age between 18 and 75 years and patients had to be scheduled for implant removal which was placed after trauma. Exclusion criteria were an active infection and/or fistula, placement of new material during the same procedure, active use of antibiotics or an immunosuppressant, a contraindication for the use of cefazolin [allergy, estimated glomerular filtration rate <35 according to the chronic kidney disease Epidemiology Collaboration equation (CKD-EPI) (20), pregnancy], and insufficient understanding of the Dutch or English language.

### Description of experiment, treatment, or surgery

All included patients received a single directly infused bolus of 2 g of cefazolin diluted in 20 cc of NaCl as a prophylactic antibiotic. The cefazolin was administered via an intravenous line within 1 hour of the start of the surgical procedure.

### Outcome measures and data collection

The primary outcomes were the cefazolin concentrations in venous plasma, target-site plasma, and target-site subcutaneous tissue. Secondary outcomes were the influences of several predictor variables on the cefazolin concentration. We registered time of administration of the dose of cefazolin and time of sample collection by hand at the operation room. Furthermore, included patients received a survey they could fill in via Castor EDC (Amsterdam, North-Holland, Netherlands) prior to the surgery as for their participation in the aforementioned WIFI-2 study. The following data were collected: age, gender, length, weight, smoking status, alcohol consumption, drug usage, and history of diabetes mellitus or peripheral arterial disease. Four additional surveys were sent at 2 weeks, 6 weeks, 3 months, and 6 months postoperatively with questions regarding functionality and and wound healing. Smoking and illegal drug abuse were defined as usage on a daily basis. Alcohol consumption was defined as an intake of at least two units per day. SSIs were classified in superficial and deep infections using the CDC criteria (21).

### Sample collection

The venous plasma samples were collected at the upper extremities by means of a standard blood sample. Target-site plasma samples were collected by collecting blood samples out of the surgical wound with a syringe and stored in an EDTA tube. Target-site tissue was collected by a subcutaneous tissue biopsy (±12.5 mm^3^) of the surgical wound and stored in a sterile container. After collection, the samples were brought to the hospital pharmacy laboratory within 1 hour and the plasma and tissue samples were stored in a −80°C freezer until sample analysis was performed. Samples were obtained on varying time points during the surgical procedure in order to obtain an impression of the time profile of cefazolin concentration. A minimum of one and a maximum of two measurements were performed per sample site per patient. Blank (target-site) plasma and tissue samples were obtained from participants of the WIFI-2 study who received a placebo (20 cc 0.9% NaCl).

### Cefazolin assay

Cefazolin plasma and tissue concentration were determined by using liquid chromatography-mass spectometry (LC-MS/MS) with electrospray ionization in the positive ionization mode on a Shimadzu LC-30 (Nishinokyo-Kuwabaracho, Japan) system coupled to an ABSciex (Framingham, MA, USA) 5500 Qtrap MS. For tissue analysis, the tissue sample (max. 400 mg) was weighted and put in a MagNA lyser green breads cup (Roche Diagnostics, GmbH, Germany) with 500 µL ultrapure water. The cups were running 60 s at 6,500 RPM in the MagNA lyser. To 20 µL of the tissue solution or plasma, 750 µL of acetonitrile/methanol 84:16 (vol/vol %) containing the internal standard cefazolin-^13^C_2_,^15^N was added to precipitate proteins. Samples were vortexed, stored at −20°C for 10 minutes to optimize protein precipitation, vortexed again, and centrifuged for 5 minutes at 2,750 × *g*. Also, 0.5 µL was injected for analysis of cefazolin onto a Thermo Scientific HyPurity aquastar 100 × 2.1 mm, 5 µm column. A chromatographic gradient was applied using eluent A (0.1% formic acid and 0.05% ammonium formate in ultrapure water) and eluent B [0.1% formic acid and 0.05% ammonium formate in ACN:MeOH (75:25 vol/vol %)]. The flow rate was 0.4 mL/min and the column oven was kept at 30°C. Using multiple reaction monitoring, cefazolin is measured in positive polarity mode as [M + H]+, using the mass transitions 458 and 326 for cefazolin-^13^C_2_,^15^N. The method for plasma samples was validated over a plasma concentration range of 1.00–100 µg/mL, including a 10-fold dilution of concentrations above 100 µg/mL. Accuracy in plasma ranged from 96.3% to 110%, intra-day precision was ≤3.0% and inter-day precision was ≤2.5%. Tissue samples were quantified based on the validated plasma concentration standards, and cross-matrix validation was performed. The accuracy of six different tissue solution samples at minimum quantifiable level was between 99.1% and 101.0%.

### Other methods

The concentrations are expressed in µg/mL for both venous and target-site plasma samples and in µg/mg for target-site tissue samples. The MIC_90_ of cefazolin for the organisms of interest are also expressed in µg/mL. The protein binding of cefazolin has to be kept in mind, as the measured concentrations are a representation of the total concentration cefazolin and not only the free and therefore active, unbound, cefazolin. The systematic review of Jongmans et al. (22) showed that the protein binding of cefazolin in plasma ranges between 55% and 87% depending on the study population (22). As for this study, the arbitrary cutoff value of 80% protein binding was chosen as this elective procedure is mostly performed in healthy patients. The same cutoff value was chosen for the target-site tissue samples, as there is no consensus regarding the binding of antibiotics in tissue yet.

### Statistical analysis and study size

The statistical analyses were done using IBM SPSS Statistics 28.0.0.1. Normality was tested using the Kolmogorv-Smirnov test and normally distributed data are shown with mean and standard deviation (SD), while non-normally distributed data are shown with median and inter-quartile range (IQR). We used linear regression for the univariate analysis in order to identify possible predictor variables for the cefazolin concentration. A backward multiple linear regression was performed when two or more possible predictor variables (*P* < 0.1) were identified in the univariate analysis. Moreover, binary logistic regression analysis was conducted to ascertain whether the cefazolin concentration served as a predictive factor for the occurrence of deep SSI. A two-sided *P*-value of <0.05 was considered significant.

## RESULTS

In total, 27 patients who received a single dose of 2 g of prophylactic cefazolin between May 2021 and March 2023 were included in this study. The patient characteristics are shown in Table 1. Of the 143 samples, five samples (three target-site tissue and two venous plasma samples) in five different patients were deemed invalid as no traces of cefazolin were measured while other samples of the same sample location of these patients did show measurable concentrations. Therefore, a total of 138 samples were included in the analysis. Follow-up was available of all patients.

**TABLE 1:** Patient characteristics

Characteristic	Value
Age in years, median (IQR)	42 (29–59)
Gender, *n* (%)	
Male	17 (63.0)
Female	10 (37.0)
Weight in kg, mean (SD)	78.77 (14.24)
Body mass index, median (IQR)	24.88 (21.76–28.70)
Substance abuse	
Smoking, *n* (%)	7 (25.9)
Alcohol, *n* (%)	4 (14.8)
Drugs, *n* (%)	3 (11.1)
Comorbidities	
Diabetes, *n* (%)	1 (3.7)
Peripheral arterial disease, *n* (%)	0 (0.0)
Location of surgery, *n* (%)	
Proximal tibia	8 (29.6)
Ankle	5 (18.5)
Hindfoot	7 (25.9)
Midfoot	7 (25.9)
Use of tourniquet, *n* (%)	0 (0.0)

**Fig 1:**
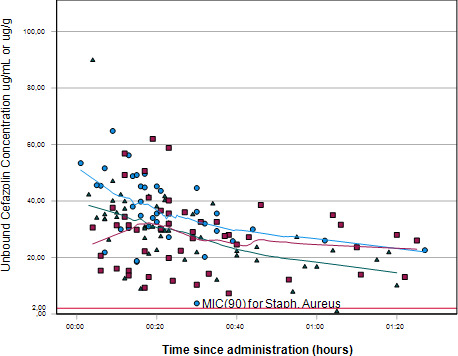
Cefazolin concentration in venous plasma (blue circle), target-site plasma (green triangle), and target-site tissue (red square). The lines represent the corresponding smooths for illustration only.

**Fig 2:**
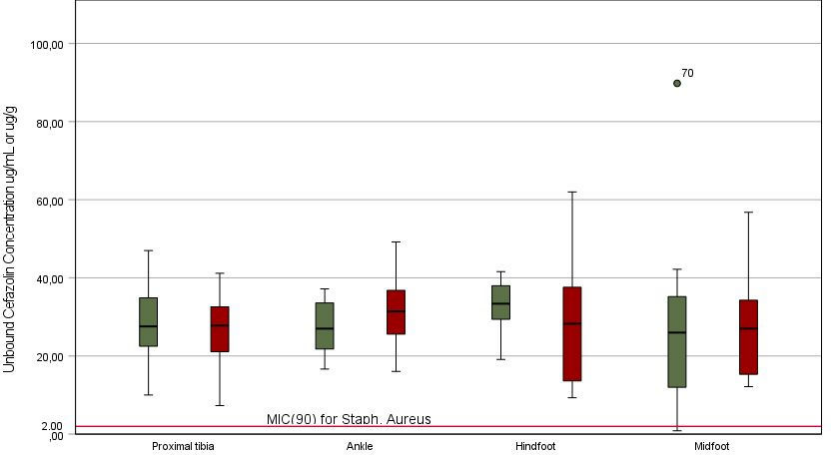
Cefazolin concentration in target-site plasma (green) and target-site tissue (red) at different locations. The box presents the second and third quartiles.

The mean concentrations of cefazolin are shown in Table 2 and the concentrations have been plotted against time in Fig. 1. Additionally, the concentrations by sample location are visualized in Fig. 2. The cefazolin concentrations between the different locations of surgery did not differ significantly in both target-site plasma and target-site tissue (*P* = 0.822 and *P* = 0.840, respectively). The mean cefazolin concentration was 2.3% higher in target-site plasma compared to target-site tissue. In all but one sample, the concentrations were above the MIC_90_ of *Staphylococcus aureus*.

**TABLE 2:** Concentration measurements

	Samples, *n*	Total concentration, mean (SD)	Unbound concentration, mean (SD)	Time since administration in minutes, median (IQR)
Venous plasma	37	181 (63) µg/mL	36 (13) µg/mL	17 (13–30)
Target-site plasma	52	145 (66) µg/mL	29 (13) µg/mL	21 (12–39)
Proximal tibia	15	144 (51) µg/mL	28 (10) µg/mL	31 (10–45)
Ankle	10	136 (34) µg/mL	27 (7) µg/mL	19 (9–56)
Hindfoot	13	159 (38) µg/mL	32 (8) µg/mL	22 (14–28)
Midfoot	14	138 (111) µg/mL	28 (22) µg/mL	16 (6–39)
Target-site tissue	49	141 (65) µg/g	28 (13) µg/g	23 (14–38)
Proximal tibia	16	132 (46) µg/g	26 (9) µg/g	36 (23–69)
Ankle	7	158 (54) µg/g	32 (11) µg/g	17 (10–46)
Hindfoot	14	145 (85) µg/g	29 (17) µg/g	23 (14–29)
Midfoot	12	140 (72) µg/g	28 (14) µg/g	17 (7–34)

Age, gender, weight, body mass index, smoking, drug usage, and diabetes did not significantly affect the cefazolin concentration in venous plasma (*P* = 0.160, *P* = 0.584, *P* = 0.479, *P* = 0.397, *P* = 0.127, *P* = 0.340, and *P* = 0.167, respectively). Also in target-site plasma, these factors did not significantly affect cefazolin concentration (*P* = 0.947, *P* = 0.200, *P* = 0.837, *P* = 0.266, *P* = 0.410, *P* = 0.339, and *P* = 0.637, respectively). This was also the case in target-site tissue (*P* = 0.431, *P* = 0.223, *P* = 0.277, *P* = 0.707, *P* = 0.196, *P* = 0.135, and *P* = n/a, respectively). Although alcohol consumption did not significantly influence the cefazolin concentration in venous plasma and target-site plasma (*P* = 0.060 and *P* = 0.090, respectively), it did significantly influence the tissue concentration (B = 8.520, SE = 5.600, and *P* = 0.029, respectively).

Four patients (14.8%) developed SSI, of which one developed a deep infection. The mean cefazolin concentration in venous plasma, target-site plasma, and target-site tissue were not significantly different compared to patients without infection (40 ± 14 vs 35 ± 12 µg/mL, 31 ± 7 vs 28 ± 14 µg/mL, and 34 ± 17 vs 27 ± 12 µg/g) (*P* = 0.346, *P* = 0.636, and *P* = 0.249, respectively).

## DISCUSSION

The primary aim of this study was to measure target-site cefazolin concentrations at different locations below the level of the knee in both tissue and target-site plasma. Concentrations in target-site plasma and target-site tissue were 25.3% and 28.2% lower than concentrations in plasma but all well above the MIC_90_ of *S. aureus*. No differences in target-site concentrations were detected between the different sample site locations.

In the Netherlands, SSIs subsequent to lower extremity surgeries are primarily attributed to gram-positive methicillin-susceptible *Staphylococcus aureus* and β-hemolytic streptococci. The epidemiological cutoff value, i.e., the highest MIC value of *S. aureus* isolates that have no acquired resistance mechanism, is 2 µg/mL, which is also the MIC_90_ for *S. aureus* (23, 24) while the MIC_90_ of 0.12 µg/mL for *Streptococcus pyogenes* is even lower (23). In the Netherlands, 98% of *S. aureus* strains are susceptible to methicillin (25). In order to be deemed efficacious as a prophylactic agent, an antibiotic should ideally maintain unbound concentrations above the MIC for at least the duration spanning from the commencement of surgical wound exposure to its closure (16). In our study, the mean unbound concentrations of cefazolin were significantly elevated in both venous plasma and target-site plasma, as well as target-site tissue, and consistently surpassed the MIC_90_ for *S. aureus* for a duration of at least 80 minutes. This conforms with international consensus which advises to administer an additional dose when surgery exceeds two times the half-life of the drug, which is approximately 240 minutes for cefazolin (26).

Several studies investigating tissue concentrations of cefazolin have been performed. In studies investigating bone samples, mean concentrations between 0.64 µg/g and 88 µg/g were found (17). However, it is our belief that SSIs originate out of the subcutaneous tissue, and subcutaneous tissue concentrations of cefazolin are therefore of more importance than bone concentrations. Currently, there are no studies investigating the cefazolin concentrations in subcutaneous target-site tissue below the level of the knee after a gift of 2 g prophylactic cefazolin. This makes this study unique of its kind and therefore difficult to compare. However, there is a recent study focusing on cefazolin target-site concentrations following open tibia or fibula fractures (27). Bates et al. administered a microcatheter in the traumatic wound to measure target-site concentrations at different moments (every hour, up to 8 hours following administration of 2 g cefazolin). This is closely related to our method and therefore comparable to our target-site tissue concentration. They found that the time the cefazolin concentration exceeded the MIC was 100%, with the lowest cefazolin concentration 30 minutes before administration of a new dose (measurement after 7.5 hours). The lowest concentration was 10.00 µg/mL. These high concentrations are in line with our results, although our measurements are more frequent in the first hour following administration.

Furthermore, the study of Bhalodi et al. (28) investigated the cefazolin concentrations in the lower extremities after admission of 1 g cefazolin (28). They found a tissue/plasma penetration ratio of 1.06 ± 0.78 which initially appears incomparable to our findings. Our measurements show that the concentrations between plasma and tissue samples are indistinguishable. However, when looking at the time range at which the samples were collected, a big difference is seen with our study. The majority of our samples were collected within 1 hour after administration, possibly explaining the disparity the study of Bhalodi et al. and our study.

As depicted in Fig. 1, a concentration below the MIC_90_ for *S. aureus* was identified in a singular target-site plasma sample. The mentioned sample was sourced from a healthy male subject aged 20 years, who did not suffer a postoperative SSI. Additionally, as mentioned in the results section, five samples were deemed invalid as no traces of cefazolin were found while other samples of the same sample location of these patients did show measurable concentrations. Except for the likelihood of a sampling or measurement error, no plausible rationales for the diminished concentration could be ascertained.

A correlation between the various sampling locations and the corresponding concentrations was not found. Previous literature has demonstrated a variation in concentrations when measured at the hip and knee levels (17, 29–31). A plausible explanation for the observed disparity could be attributed to the differences in sampling time intervals. Specifically, the samples obtained at the proximal tibia were collected at a median time of 31 and 36 minutes, whereas measurements at the height of the midfoot were taken at a median time of 16 and 17 minutes. This interval is the moment following administration in which the concentration dispersion is the largest. Consequently, a valid comparison between the concentrations at these two locations could not be established due to the decline in concentrations over time.

A remarkable result was the positive influence of an alcohol intake of ≥2 units a day on the cefazolin concentration in target-site tissue, with a mean target-site tissue concentration which was 41.1% higher (37.40 ± 11.24 vs 26.51 ± 12.71 µg/g). However, the small sample size of this subgroup has to be kept in mind. Additionally, the clinical relevance remains limited, as the mean concentrations of both groups are well above the MIC_90_ for the relevant microorganisms.

Of the included patients, four developed a SSI. Among these cases, three were categorized as superficial infections, while the remaining case met the CDC criteria of a deep infection. Subsequent bacterial culture analysis of the deep infection revealed the presence of methicillin-susceptible *Staphylococcus aureus*. Interestingly, this patient also developed a deep SSI after the primary surgery, where the material was placed. The identical bacterial strain was identified on both occasions, suggesting the presence of an enduring low-grade infection, and subsequently becoming clinically evident subsequent to the surgical procedure involving the removal of the implant. This patient was not excluded, as signs of active infection were not present during surgery. Prompt and appropriate medical intervention was initiated, involving a regimen of surgical debridement and intravenous administration of antibiotics, after which he fully recovered. The other three patients were successfully treated with oral antibiotics.

As mentioned by Jager et al. (32), reliable information regarding the tissue penetration of antibiotics is scarce, as well as the relation with clinical outcome (32). The results of our study show that the target-site plasma concentration is almost identical to the target-site tissue concentration, and we did not find a lower concentration of cefazolin in the patients who developed a SSI.

The pursuit of the optimal prophylactic antibiotic agent and its appropriate dosage remains a subject of ongoing investigation. Insights from the WIFI-1 study have indicated that the administration of 1 g of cefazolin, prior to the removal of implants below the knee level, does not yield a reduction in the incidence of SSIs (33). The WIFI-2 study, which investigates the efficacy of the admission of 2 g of cefazolin instead of 1 g, is conducted at the moment of writing this paper (19). The results of the WIFI-2 study should give decisive evidence regarding the efficacy of cefazolin in implant removal surgery below the level of the knee. Furthermore, the microorganisms causing SSIs should continuously be monitored in order to make sure that they are still covered by the given antibiotic prophylaxis.

We believe the results of our primary outcome are reliable, given the used method, amount of included patients, and the sample size. A limitation, however, is the incomparability of the concentrations between the different sample locations due to the varying sample intervals. Additionally, the sample sizes of the subgroups were small and this should be taken into account when placing the results into perspective.

In conclusion, 2 g of prophylactic cefazolin demonstrates adequacy in maintaining coverage for a duration of at least 80 minutes of surgery below the level of the knee, significantly surpassing the MIC_90_) required to combat the most prevalent microorganisms. Notably, no differences in cefazolin concentrations were observed among the diverse sampling locations at the knee and below. Finally, it is worth highlighting that this study represents the first investigation of its kind to assess cefazolin concentrations in the distal lower extremities by examining both plasma and tissue samples in this magnitude.
